# Response inhibition signals and miscoding of direction in dorsomedial striatum

**DOI:** 10.3389/fnint.2012.00069

**Published:** 2012-09-07

**Authors:** Daniel W. Bryden, Amanda C. Burton, Vadim Kashtelyan, Brian R. Barnett, Matthew R. Roesch

**Affiliations:** ^1^Department of Psychology, University of Maryland, College ParkMD, USA; ^2^Program in Neuroscience and Cognitive Science, University of Maryland, College ParkMD, USA

**Keywords:** dorsal striatum, stop-signal, inhibition, behavioral control, single unit, rat

## Abstract

The ability to inhibit action is critical for everyday behavior and is affected by a variety of disorders. Behavioral control and response inhibition is thought to depend on a neural circuit that includes the dorsal striatum, yet the neural signals that lead to response inhibition and its failure are unclear. To address this issue, we recorded from neurons in rat dorsomedial striatum (mDS) in a novel task in which rats responded to a spatial cue that signaled that reward would be delivered either to the left or to the right. On 80% of trials rats were instructed to respond in the direction cued by the light (GO). On 20% of trials a second light illuminated instructing the rat to refrain from making the cued movement and move in the opposite direction (STOP). Many neurons in mDS encoded direction, firing more or less strongly for GO movements made ipsilateral or contralateral to the recording electrode. Neurons that fired more strongly for contralateral GO responses were more active when rats were faster, showed reduced activity on STOP trials, and miscoded direction on errors, suggesting that when these neurons were overly active, response inhibition failed. Neurons that decreased firing for contralateral movement were excited during trials in which the rat was required to stop the ipsilateral movement. For these neurons activity was reduced when errors were made and was negatively correlated with movement time suggesting that when these neurons were less active on STOP trials, response inhibition failed. Finally, the activity of a significant number of neurons represented a global inhibitory signal, firing more strongly during response inhibition regardless of response direction. Breakdown by cell type suggests that putative medium spiny neurons (MSNs) tended to fire more strongly under STOP trials, whereas putative interneurons exhibited both activity patterns.

## Introduction

The ability to inhibit action is critical for everyday behavior and is disrupted in several diseases including attention deficit hyperactivity disorder, schizophrenia, substance abuse, pathological gambling, Tourette syndrome, Parkinson's Disease, and obsessive-compulsive disorder (Schachar et al., 1995; Oosterlaan and Sergeant, 1998; Oosterlaan et al., 1998; Rubia et al., 1998, 2005, 2007; Fillmore and Rush, 2002; Gauggel et al., 2004; Aron and Poldrack, 2005; Kalanithi et al., 2005; Monterosso et al., 2005; Nigg et al., 2005; Bellgrove et al., 2006; Fillmore et al., 2006; Schachar et al., 2007; Durston et al., 2009; Eagle and Baunez, 2010; Kataoka et al., 2010; Leventhal et al., 2012). Although dorsal striatum has been implicated in habitual, over-learned, and automatic behaviors (Miyachi et al., 1997; Jog et al., 1999; Matsumoto et al., 1999; Graybiel, 2000; Bailey and Mair, 2006; Yin and Knowlton, 2006; Schmitzer-Torbert and Redish, 2008), recent work has pointed to the mDS as being part of a circuit that is also involved in response inhibition (Eagle and Baunez, 2010). Unfortunately, little is known about the neural signals in mDS that are involved in preventing unwanted behavior.

Pharmacological and anatomical studies have demonstrated that mDS is involved in response inhibition (Eagle and Baunez, 2010), but its exact role in this critical function remains elusive. For example, during performance of a stop-signal task in which rats had to stop an ongoing movement in the minority of trials, rats showed reduced ability to inhibit responding after mDS lesions (Eagle and Robbins, 2003; Eagle and Baunez, 2010). In this task, rats were required in the large majority of trials (80%) to respond quickly to an instrumental stimulus (light). On 20% of trials, rats were signaled by a tone to “stop” sometime between the initiation of the response and its final execution. Stopping was easier when the stop-signal (tone) came on earlier as opposed to immediately before the instrumental response (lever press). Rats with mDS lesions needed earlier warnings to be able to adequately inhibit movement as compared to controls suggesting a deficit in response inhibition. Unfortunately, this result was tainted by the finding that rats were also slower on non-STOP trials (i.e., GO-trials), making the pure response inhibition interpretation a difficult one.

Similarly intriguing results have been found in other tasks. For example, in the 5 choice serial reaction time task, rats with DS lesions were unable to refrain from action before the appropriate time (Rogers et al., 2001; Christakou et al., 2004; Eagle and Baunez, 2010). During performance of this task, rats responded to a brief visual stimulus after a fixed or variable interval (e.g., ~5 s). Responses made prior to the end the delay period were considered premature errors and were more common in rats with DS lesions. Although this result suggests that response inhibition is dependent on DS, others have failed to report premature responding after DS lesions during performance of similar tasks (Brown and Robbins, 1989; Hauber and Schmidt, 1994; Eagle and Baunez, 2010).

This mixed bag of results likely reflects that different populations of neurons in mDS are performing different functions and that global destruction of mDS is not sufficient to understand its role in behavioral inhibition. This work points to the need for a single unit recording study in rats to examine the neural mechanism by which mDS promotes and inhibits behavior. Further, to better understand what goes wrong in disorders that impact impulse control, we first need to elucidate what neural signals give rise to behavior. To address these issues we devised a modified version of tasks commonly used in clinical studies to assess the ability to stop an ongoing action. This task requires rats in the minority of trials (20%) to stop an ongoing instrumental response and redirect behavior to the opposite direction. Rats were slow on these trials and often failed to inhibit behavior demonstrating that we were tapping into response inhibition mechanisms. We found signals related to response inhibition and the miscoding of direction on STOP trials in which rats had to cancel movement and redirect behavior.

## Methods

### Subjects

Male Long–Evans rats were obtained at 175–200 g from Charles River Labs. Rats were tested at the University of Maryland in accordance with NIH and IACUC guidelines.

### Surgical procedures and histology

Surgical procedures followed guidelines for aseptic technique. Electrodes were manufactured and implanted as in prior recording experiments. Rats had a drivable bundle of 10 25-μm diameter FeNiCr wires (Stablohm 675, California Fine Wire, Grover Beach, CA) chronically implanted in the left or right hemisphere dorsal to mDS (*n* = 7 rats; 0.4 mm posterior to bregma, 2.4 mm left (*n* = 3) or right (*n* = 4) of midline, and 3.5 mm ventral to the brain surface). Immediately prior to implantation, these wires were freshly cut with surgical scissors to extend ~1 mm beyond the cannula and electroplated with platinum (H_2_PtCl_6_, Aldrich, Milwaukee, WI) to an impedance of ~300 kOhms. Cephalexin (15 mg/kg p.o.) was administered twice daily for two weeks post-operatively to prevent infection.

### Behavioral task

Recording was conducted in aluminum chambers approximately 18″ on each side with downward sloping walls narrowing to an area of 12″ × 12″ at the bottom. On one wall, a central odor port was located above two adjacent fluid wells. Directional lights were located next to fluid wells. House lights were located above the panel. Task control was implemented via computer. Port entry and licking was monitored by disruption of photobeams.

**Figure 1:**
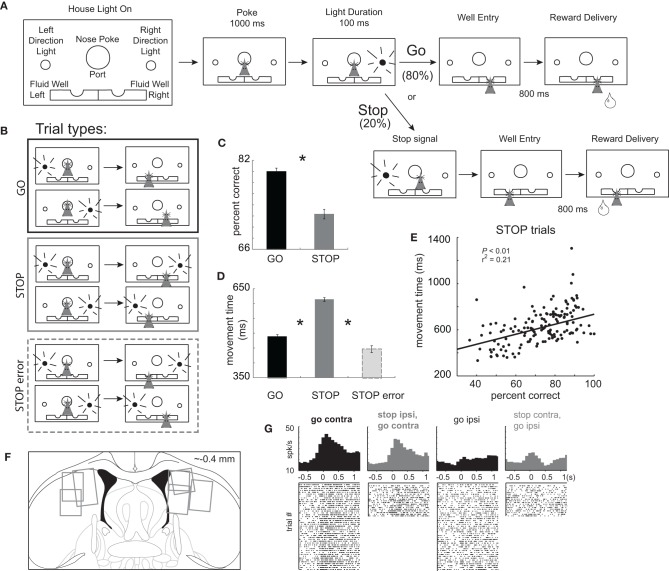
**Task Design. (A)** House lights signaled the rat to nose poke into the center port and wait 1000 ms before one of two directional lights were illuminated for 100 ms, instructing the rat to respond to either the left or right fluid well. On 20% of trials, upon port exit, the light opposite of the first light turned on to tell the rat to stop the current action and respond in the opposite direction (the direction of the second cue light). After entering the correct fluid well rats were required to wait 800 ms before reward delivery. **(B)** There were two basic conditions, stop and go, by two directions. We refer to direction as being contralateral or ipsilateral to the recording location. **(C)** Percent correct scores as a function of all trials in which a choice was made to one of the fluid wells. **(D)** Latency to move from the nose poke port to the well. **(E)** Correlation between movement time and percent correct scores during performance of STOP trials. Asterisks: planned comparisons revealing statistically significant differences (*t*-test, *p* < 0.05). **(F)** Location of recording sites. Gray boxes mark extent of recording sites. **(G)** Single cell example of a neuron aligned on port exit that fires more strongly under GO than STOP trials made in the contralateral direction. Overall this neuron fired more strongly for movement ultimately made in the contralateral direction, which we refer to as a “directional” signal. The directional signal (contra minus ipsilateral movement) is weaker for STOP compared to GO trials.

The basic design of a trial is illustrated in Figures 1A,B. Each trial began by illumination of house lights that instructed the rat to nose poke into the central port. Nose poking began a 1000 ms pre-cue delay period. At the end of this delay, a directional light to the animal's left or right was flashed for 100 ms. The trial was aborted if a rat exited the port at any time prior to offset of the directional cue light. On 80% of trials, presentation of either the left or right light signaled the direction in which the animal could respond in order to obtain sucrose reward in the fluid well below. On 20% of trials, once the rat exited the nose poke port, the light opposite to the location of the originally cued direction turned on and remained on until the behavioral response was made. These trials were randomly interleaved with GO trials. Rats were required to stop the movement signaled by the first light and respond in the direction of the second light. After correct responses, rats had to remain in the well for 800 ms (pre-fluid delay) before reward delivery (10% sucrose solution). Trials were presented in a pseudorandom sequence such that left and right trials were presented in equal numbers (±1 over 250 trials). The intertrial interval (ITI) was 4 s.

### Single-unit recording

Procedures were the same as described previously (Bryden et al., 2011). Wires were screened for activity daily; if no activity was detected, the rat was removed, and the electrode assembly was advanced 40 or 80 μm. Otherwise active wires were selected to be recorded, a session was conducted, and the electrode was advanced at the end of the session. Neural activity was recorded using two identical Plexon Multichannel Acquisition Processor systems (Dallas, TX), interfaced with odor discrimination training chambers. Signals from the electrode wires were amplified 20X by an op-amp headstage, located on the electrode array. Immediately outside the training chamber, the signals were passed through a differential pre-amplifier (Plexon Inc, PBX2/16sp-r-G50/16fp-G50), where the single unit signals were amplified 50X and filtered at 150–9000 Hz. The single unit signals were then sent to the Multichannel Acquisition Processor box, where they were further filtered at 250–8000 Hz, digitized at 40 kHz and amplified at 1–32X. Waveforms (>2.5:1 signal-to-noise) were extracted from active channels and recorded to disk by an associated workstation with event timestamps from the behavior computer. Waveforms were not inverted before data analysis.

### Data analysis

Units were sorted using Offline Sorter software from Plexon Inc. (Dallas, TX), using a template matching algorithm. Sorted files were then processed in Neuroexplorer to extract unit time-stamp and relevant event markers. These data were subsequently analyzed in Matlab (Natick, MA). Baseline firing was taken during a 1 s epoch starting 2 s prior to trial initiation (nose poke). Average interspike intervals (ISIs) were computed during the entire recording session for each neuron. For the majority of the analysis, activity was examined during the period between nose poke exit and well entry (response epoch), while the movement was being made and/or canceled. Wilcoxon tests were used to measure significant shifts from zero in distribution plots (*p* < 0.05). *T*-tests or ANOVAs were used to measure within cell differences in firing rate (*p* < 0.05). Pearson Chi-square tests (*p* < 0.05) were used to compare the proportions of neurons.

## Results

Rats were trained on a task in which spatial cue lights instructed the direction of the behavioral response necessary to obtain reward. The sequence of events is illustrated in Figure 1A. House lights indicated the start of the trial. Rats began the trial by nose poking into the central port. After 1000 ms, one of two lights (left or right) was illuminated for 100 ms. During 80% of trials, the rule was to detect the light and make a behavioral response in that direction. These trials will be referred to as “GO” trials. On the remaining 20% of trials, immediately after the rat exited the nose poke port, a second light was flashed, opposite to the first light, indicating that the rat must inhibit their initial action and respond to the opposite well (i.e., in the direction of the second light). These trials will be referred to as “STOP-change” or “STOP” trials for short. The STOP cue was illuminated only after the movement had been initiated, thus we are examining the rats ability to inhibit a behavior already set in motion. For all trials reward was delivered 800 ms after entering the fluid reward well. There were a total of four trial-types: go-left, go-right, stop-left-go-right, and stop-right-go-left (Figure 1B), however, for the remainder of the paper, response direction (i.e., left and right) will be referenced to the location of the recording site (contralateral or ipsilateral).

Inhibition and redirection of the behavioral response necessary to perform STOP trials resulted in significantly slower movement speeds from port exit to well entry and reduced accuracy compared to GO trials. (Figures 1C and D; *t*-test; percent correct: *t*_(436)_ = 11.0, *p* < 0.01; movement time: *t*_(436)_ = 34.7, *p* < 0.01). Slower latencies resulted in better task performance consistent with a speed accuracy trade off. This is illustrated in Figure 1E which plots movement times (well entry minus port exit) on STOP trials for each recording session. During sessions in which the rat was slower, performance was better (*p* < 0.05; *r*^2^ = 0.21). Consistent with this finding, STOP trial error movement times were significantly faster than movement times on correctly performed STOP trials (Figure 1D; *t*_(375)_ = 25.1, *p* < 0.01). These results demonstrate that rats were planning and generating a movement prior to illumination of the STOP signal, in response to illumination of the first cue light.

Use of this task in the context of behavioral neurophysiology allows us to examine activity related to response inhibition and redirection of behavior. Trials during which the movement had to be stopped and redirected will be directly compared to responses made in the same direction, which could not be done with more typical stop-signal tasks. After illumination of the STOP-change cue it took animals 140 ms to stop and redirect behavior as computed by subtracting the movement time on GO trials from the movement time on STOP trials averaged over all recording sessions. We will refer to this time as the “stop change reaction time” or “SCRT.” As we will show below, activity changes related to stopping and redirecting behavior preceded the SCRT.

### Activity related to contralateral movement was modulated by GO and STOP trial-types

We recorded 437 mDS neurons in seven rats from the recording locations illustrated in Figure 1F. Previous reports have shown that mDS is critical for acquisition and expression of cue-guided responses and that activity in mDS is strongly associated with movement, in particular movement contralateral to recording and lesion/inactivation locations (Cook and Kesner, 1988; Schultz and Romo, 1988; Brown and Robbins, 1989; Carli et al., 1989; Wiener, 1993; Mink, 1996; Packard and McGaugh, 1996; Brasted et al., 1997; Redgrave et al., 1999; Ragozzino et al., 2001, 2002a,b; Schmitzer-Torbert and Redish, 2004, 2008; Yeshenko et al., 2004; Barnes et al., 2005; Hikosaka et al., 2006; Lau and Glimcher, 2007; Samejima and Doya, 2007; Kimchi and Laubach, 2009a,b; Kimchi et al., 2009; Bromberg-Martin et al., 2010; Gage et al., 2010; Stalnaker et al., 2010; van der Meer et al., 2010). Consistent with these results we found that many neurons were modulated by response direction. This is illustrated in Figure 1G, which plots the average firing of a single mDS neuron. This neuron showed a strong directional signal, firing more strongly on contralateral GO trials compared to ipsilateral GO trials (black).

We hypothesized that contralateral motor signals such as these would be modulated by trial-type, STOP or GO, as in the single neuron example. For this neuron, firing was stronger for GO vs. STOP trials for movements ultimately made in the contralateral direction. Thus, for our first analysis we asked how many neurons fired significantly more or less strongly for successful GO compared to successful STOP trials when the rat responded to the fluid well contralateral to the recording site (response epoch = port exit to well entry; *t*-test; *p* < 0.05). Then, we further characterize activity of these neurons by examining directional selectivity (contra- vs. ipsilateral) on GO and STOP trials independently, and determined how firing differed when responses were not correctly inhibited on STOP trials.

Of the 437 mDS neurons, 81 (19%) showed a significant difference between STOP and GO trials during contralateral movement. Thirty-four of these fired significantly more strongly for GO vs. STOP trials, whereas 47 showed significantly stronger firing on STOP compared to GO trials. This proportion is more than expected by chance alone (chi-square = 168; *p* < 0.01) and the frequency of neurons showing increases for GO vs. STOP trials during contralateral movement was not significantly different (34 vs. 47; chi-square = 2.1; *p* = 0.15). As we will describe below, both populations of neurons were directionally selective as defined by differential firing between contralateral and ipsilateral GO trials, and showed changes in firing dependent on whether or not movements were correctly inhibited.

### Neurons that showed elevated firing during contralateral GO trials showed reduced direction selectivity during STOP trials

**Figure 2:**
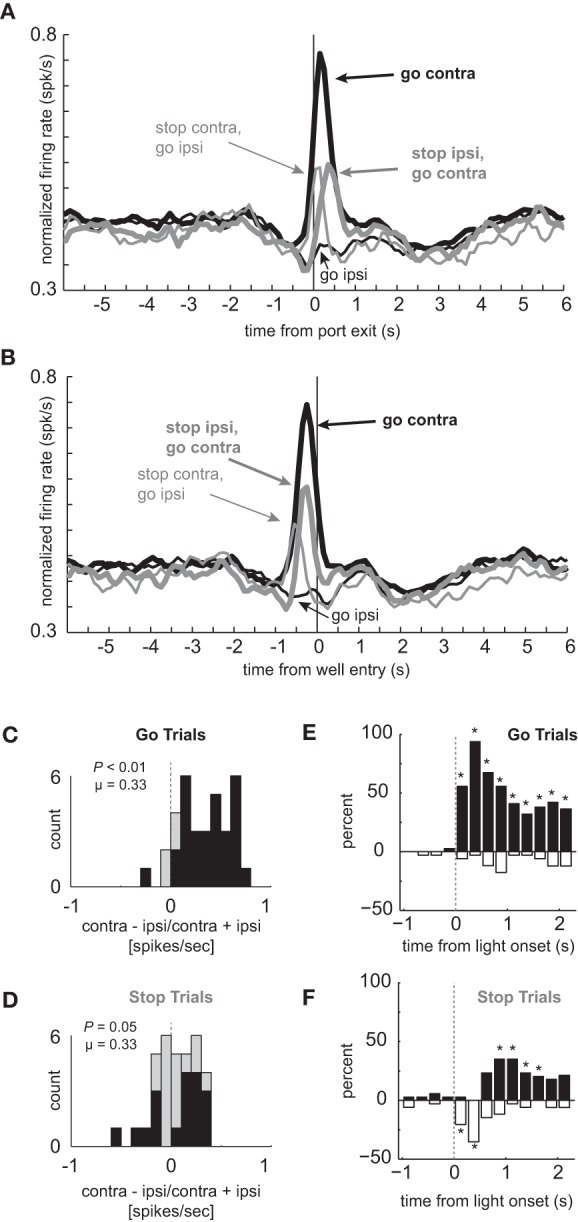
**Response inhibition and reduced directional selectivity in mDS. (A,B)** Population of neurons that showed significant elevation on GO vs. STOP trials for contralateral movements (*n* = 34). Direction is referenced to recording location. Thick and thin lines represent contralateral and ipsilateral direction, respectively. Black and gray represents GO and STOP trials, respectively. Activity is aligned to port exit **(A)** and fluid well entry **(B)**, respectively. **(C,D)** Distribution of directional indices determined by subtracting activity taken from port exit to well entry (response epoch) for ipsilateral movement trials from contralateral movement trials and dividing by the sum of the two (contra – ipsi/contra + ipsi). Black bars represent the number of neurons that showed a significant difference between contralateral and ipsilateral trial types during the response epoch (*t*-test; *p* < 0.05). **(C)** and **(D)** reflect the distribution of direction indices for GO and STOP trials, respectively. Distributions are determined to be significantly different from zero via Wilcoxon. **(E,F)** Time course of significant effects for 250 ms epochs aligned to the onset of the first directional cue light. Height of positive and negative bars reflects the percentage of neurons that fired more strongly on trials for correct movements made in the contralateral and ipsilateral movement, respectively, for GO **(E)** and STOP **(F)** trials. Asterisks represent significant differences between counts of neurons (black vs. white bar; chi-square; *p* < 0.05).

The average population histogram for neurons that showed elevated firing on contralateral GO trials compared to STOP trials is shown in Figures 2A and B (*n* = 34). As defined by the analysis and illustrated in the single cell example, activity was higher for contralateral GO trials (thick black) as compared to STOP trials (thick gray) in which the rats correctly inhibited the ipsilateral movement in order to make the contralateral one. These neurons encoded response direction, firing significantly more strongly for contralateral vs. ipsilateral movement on GO trials (thick black vs. thin black) starting after illumination of cue lights that occurred on average 362 ms (standard deviation = 115 ms) before port exit (100 ms light duration plus time from extinction of the light until port exit). This direction signal was quantified by computing an index, which reflected the difference between contralateral and ipsilateral movement (contra – ipsi/contra + ipsi) and by performing a within-neuron comparison of direction on GO trials during the response epoch (port exit to well entry; *t*-test; *p* < 0.05). The distribution was significantly shifted in the positive direction (Figure 2C; Wilcoxon; *p* < 0.01) and the counts of individual neurons that fired significantly more strongly for movement in the contralateral direction were in the large majority (Figure 2C; black bars; 29 vs. 1; chi-square = 25.9; *p* < 0.01). Directional signals emerged quickly after illumination of the first cue light and prior to port exit exhibiting significant differential firing between ipsilateral and contralateral GO trials during the 100 ms bin that preceded port exit (*t*_(33)_ = 4.01, *p* < 0.01; black thick vs. black thin). Selectivity prior to execution of the movement, during presentation of the cue light, suggests that these neurons are driving behavior in the contralateral movement.

Next, we examined activity on STOP trials. If activity of these neurons were facilitating movement in the contralateral direction, then selectivity on STOP trials might be disrupted, reflecting the slower and less accurate performance observed on this trial type (Figures 1C–E). Indeed, the directional response signal was significantly delayed and reduced on STOP trials. This can be realized by comparing the difference between thick and thin gray lines as compared to the difference between thick and thin black lines (Figures 2A and B). The temporal pattern of activity on these trials reflected the initial encoding of the movement instructed by the first light followed by the redirection of behavior in the opposite direction as directed by the second light. On STOP trials during which the contralateral movement was signaled then canceled, activity rose quickly and then dropped off before well entry (Figures 2A and B; thin gray). This likely reflects the initiation of the contralateral movement, followed by its correction. On STOP trials during which the ipsilateral movement was signaled, canceled, and redirected contralaterally, activity took longer to rise, peaking just before well entry (Figures 2A and B; thick gray). The slow development of directional signals in mDS during STOP trials (gray lines) is consistent with slower movement times observed on these trials (Figure 1D).

The directional signal indices for STOP trials during the response epoch are plotted in Figure 2D. The distribution was not significantly shifted (Wilcoxon; *p* = 0.05) and the counts of neurons showing significantly increased firing for contralateral and ipsilateral movement were not significantly different from each other (Figure 2D; black bars; 6 vs. 12; chi-square = 1.9; *p* = 0.16). Thus, there was considerable unresolved response conflict in mDS on STOP signal trials with roughly equal numbers of neurons signaling movement in each of the two directions during the response epoch.

The reduced directional signal on STOP signal trials, in part, reflects that these neurons encoded one direction and then the other. To better illustrate the timing of this response correction and the conflict that emerges between competing signals, we plotted the percentages of neurons that fired significantly more or less strongly for contralateral movement during 250 ms bins aligned to light onset for GO (Figure 2E) and STOP (Figure 2F) trials. In these plots, the height of each black and white bar indicates the percentage of neurons that fired more strongly for contralateral and ipsilateral movements, respectively. Under GO trials, starting in the first 250 ms bin after light onset, the percentage of neurons that fired significantly more strongly for contralateral movements were in the large majority and vastly outnumbered those firing more strongly for ipsilateral movement. Under STOP trials (Figure 2F), mDS encoded the wrong direction first, followed by roughly equal numbers encoding both directions, and then, finally, encoded the correct direction, which was still less than observed during equivalent times on GO trials (Figure 2E). This further illustrates the role that these neurons likely play in promoting the initial movement, shutting it down and eventually signaling for the correct behavior.

### Neurons that showed elevated firing during contralateral GO trials miscoded the correct response on errant STOP trials

**Figure 3:**
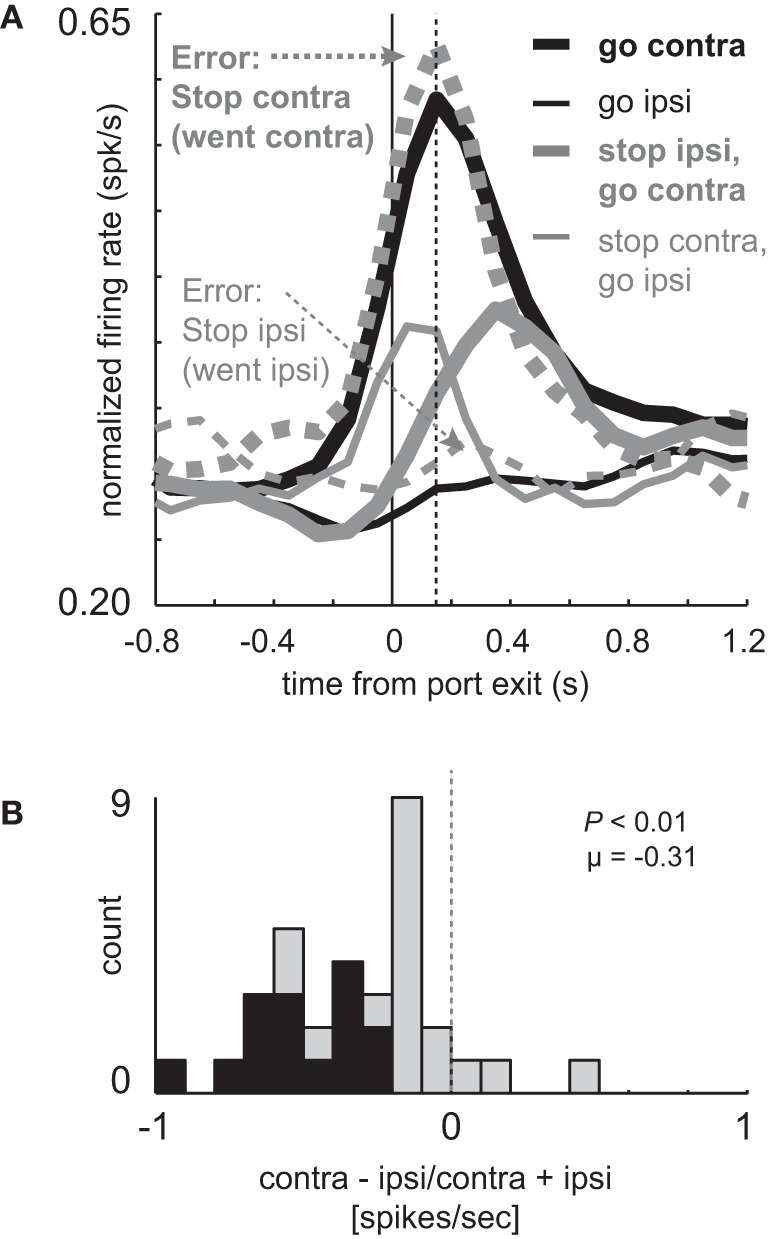
**Miscoding of directional selectivity in mDS during STOP errors. (A)** Curves representing normalized population firing rate (*n* = 33) for neurons that showed elevated firing on GO compared to STOP trials for movements made in the contralateral direction. Neurons were taken from sessions where at least one STOP trial occurred in each direction. Thick and thin lines represent contralateral and ipsilateral direction, respectively. Black and gray lines represent GO and STOP trials, respectively. Solid and dashed lines represent correct responses and errant responses on STOP trials, respectively. Thick dashed gray lines reflect a condition where the rats had to cancel the contralateral movement but failed (i.e., went contralateral). Thin gray dashed lines reflect a condition where the rats had to cancel the ipsilateral movement but failed (i.e., went ipsilateral). Vertical dashed line represents the time required to stop and redirect behavior (SCRT) as computed by taking the difference between correct STOP and GO trials during these sessions. **(B)** Distribution of directional indices for the firing of single neurons during STOP trial errors (contra – ipsi/contra + ipsi) where direction refers to the side in which the animal responded, not the side cued by the second cue light. This activity was analyzed during the response epoch (*t*-test; *p* < 0.05).

These results suggest that increased firing prior to and during the execution of movement is driving or facilitating behavior in the contralateral direction. If true, when rats make mistakes, activity should reflect the direction of the movement. To address this hypothesis we examined firing on errant STOP trials in which rats did not inhibit the movement after illumination of the STOP light (i.e., incorrectly followed the direction signaled by the first light). For this analysis, we examined sessions during which there was at least one STOP error in both directions in order to compare all of the trial-types for every cell. Activity during STOP trial errors is plotted in Figure 3A along with correct GO and STOP trials during those sessions (*n* = 33). As in the population analyzed in Figure 2A (*n* = 34), directional signals were evident for correct GO trials and reduced for correct STOP trials.

Importantly, activity changes on successful STOP trials preceded the time necessary for rats to stop and redirect behavior (SCRT) as computed by the difference between correct STOP and GO trials suggesting that changes in firing occurred before the behavior was be inhibited. For these sessions, the SCRT was 148 ms after port exit (Figure 3A; vertical dashed line), which remarkably corresponds to the time at which activity peaked on GO trials. On correct STOP trials, initially directed to the contralateral direction (thin gray), activity ceased rising before the SCRT and quickly declined after the SCRT, never reaching the strength observed on contralateral GO trials (Figure 3A; thick black). This decline was not observed on incorrect STOP trials that were erroneously generated in the contralateral direction (Figure 3A; thick gray dashed).

On STOP error trials in which the contralateral direction was not inhibited, activity was high and not statistically different from contralateral GO trials (thick black; *t*-test; *t*_(29)_ = 0.29; *p* = 0.77) rising quickly and peaking at the time of the SCRT (Figure 3A; vertical dashed line). On errant STOP trials in which the ipsilateral movement was not inhibited (thin dashed gray), activity was low and not statistically different than ipsilateral GO trials (thin black; *t*-test; *t*_(29)_ = 1.2; *p* = 0.25). As a result, the directional signal on incorrect STOP trials was the opposite of what the stop-signal cue instructed and reflected the actual movement of the rats. This is illustrated in Figure 3B which plots the directional signal index on errant STOP trials. Here, contralateral and ipsilateral refer to the direction that the animal was supposed to go, not the way they went. Up to now, the signaled and actual responses coincided with each other.

The distribution of directional signal indices were significantly shifted in the negative direction (Figure 3B; Wilcoxon; *p* < 0.01) and the number of neurons that miscoded the direction were in the majority (Figure 3B; black bars; 15 vs. 0; chi-square = 14.8; *p* < 0.01). Thus, behavioral failures of inhibition were associated with miscoding of direction in mDS.

### Neurons that showed elevated firing during contralateral GO trials exhibited stronger firing when rats made faster responses

So far our results suggest that firing in mDS is tightly correlated with response direction. Neurons such as these are thought to increase firing to a response threshold at which time the movement is generated. Consistent with this, when activity was strong, contralateral movements were erroneously generated on STOP errors (Figure 3A; dashed gray), and when contralateral mDS signals turned off in time, prior to the SCRT, the movement was canceled and the correct response generated (Figure 3A; gray).

These results suggest a relationship between firing and speed of the response in that higher firing rates during contralateral GO trials should correspond to faster movement times. Likewise, stronger signaling for contralateral movement should lead to slower responding when that movement needs to be canceled on STOP trials. To further examine the relationship between firing and motor output we divided trials into fast and slow responding for each of the four conditions individually and replotted the population histograms (Hanes and Schall, 1996). This was done by simply computing the movement time for each trial in each recording session, sorting them by speed of the response, and dividing the total trials in half. The movement times in the top and bottom half were significantly different for each of the four conditions (*t*-test; *p*'s < 0.01). Faster and slower correct GO trials had a mean movement time of 330 ms and 575 ms, respectively. For correct STOP trials, the average fast and slow trials were 489 ms and 721 ms, respectively.

**Figure 4:**
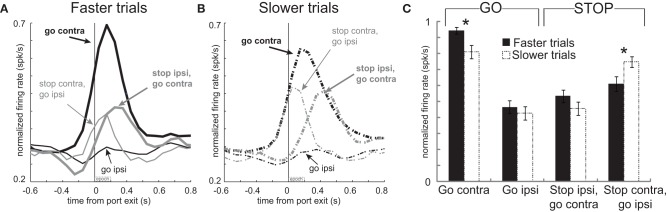
**Activity in mDS was related to movement time. (A,B)** Curves representing normalized population firing rate during performance of each of the four conditions for neurons that showed elevated firing on GO compared to STOP trials for movements made in the contralateral direction. For each condition trials were broken down by whether the behavioral response was either faster or slower. This was done by sorting trials by movement time and then by dividing trials within each condition in half. Please see text for more detail. **(C)** Bar plots allow for direct comparison. Average firing was taken 200 ms after port exit. Error bars indicate standard errors of the mean (SEM). Asterik mark indicates *p* < 0.05.

The average population histograms, split into faster and slower trials, are illustrated in Figures 4A and B, respectively. There were clear firing differences between fast and slow trials immediately after exit from the nose poke port. Just after port exit, activity was significantly stronger when rats were faster, compared to slower, for responses made on GO trials in the contralateral direction [thick solid black **(A)** vs. thick dashed black **(B)**; *t*-test; *t*_(66)_ = 2.8; *p* < 0.01]. Average firing rates during the first 200 ms after port exit, with error bars (SEM), are plotted in Figure 4C to allow for direct comparison. Although neural signals related to contralateral GO trials differed from each other, there was no significant difference between activity during fast and slow ipsilateral movements for correctly performed GO responses [thin solid black **(A)** vs. thin dashed black **(B)**] or on STOP trials when rats correctly moved in the contralateral direction [thick solid gray **(A)** vs. thick dashed gray **(B)**; *t*-test; *t*_66_'s < 1.4; *p*'s > 0.18]. However, activity was significantly higher when rats were slow to abandon the contralateral movement to respond in the ipsilateral direction [thin solid gray **(A)** vs. thin dashed gray **(B)**; *t*-test; *t*_(66)_ = 2.5; *p* < 0.02; Figure 4C: Stop contra, go ipsi]. Stronger firing when contralateral movements were faster combined with higher firing on errors suggests that phasic increases in firing of these neurons promote contralateral movement. This also explains why rats were slow to move ipsilaterally when activity was high; stronger promotion of contralateral movement would presumably slow ipsilateral movement.

### Activity related to inhibition of movement during STOP trials

Above, we have described data suggesting that a population of neurons in mDS facilitate behavior in the contralateral direction. These neurons were defined by having significantly higher firing on GO vs. STOP trials when movements were made in the contralateral direction. In this section, we perform the same analysis on neurons that showed the opposite effect; stronger firing on STOP trials redirected to the contralateral direction. Stronger firing on STOP trials in one direction suggests that they might play a role in inhibiting specific movements that might oppose the desired response.

**Figure 5:**
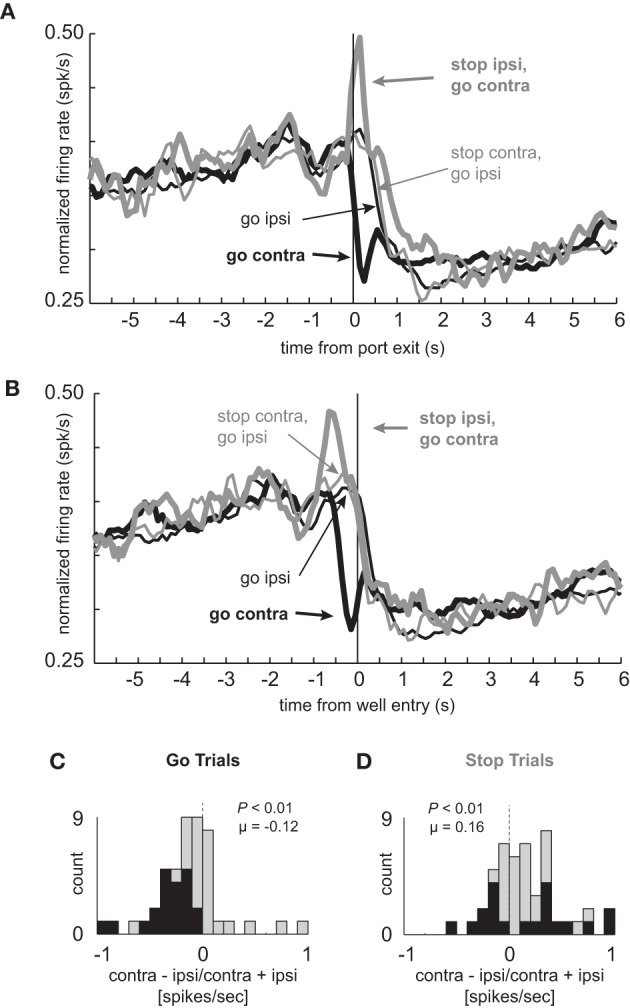
**Cells that fire more for STOP trials in the contralateral direction selectively signal the inhibition of ipsilateral movement. (A–D)** Population of neurons that showed significant elevation on STOP vs. GO trials for contralateral movements (*n* = 47). All other conventions as used in Figure 2.

Of the total 437 mDS neurons, 47 (11%) showed significantly increased firing on STOP trials relative to GO trials during the response epoch (port exit to well entry), the frequency of which is more than expected by chance alone (chi-square = 30.3; *p* < 0.05). The average population histograms for these neurons are shown in Figures 5A and B. As defined by the analysis, activity was higher on trials in which the rats successfully stopped and moved in the contralateral direction (thick gray), as compared to contralateral movements on GO trials (thick black). Activity diverged at the time of port exit, after illumination of cue lights. These cells differed dramatically from those described above in that they gradually increased firing prior to movement initiation and then phasically increased and decreased on STOP and GO trials for contralateral movement, respectively. Activity during STOP (thick gray) and GO (thick black) trials for correct responses made in the contralateral direction differentiated very quickly, showing significant differences in the 100 ms bin after (*t*-test; *t*_(46)_ = 3.62; *p* < 0.01) but not before port exit (*t*-test; *t*_(46)_ = 0.04; *p* = 0.97).

As in the population of neurons that fired more strongly under GO trials, these neurons were also highly directional in nature, due to decreased firing on contralateral but not ipsilateral movement trials (thick black vs. thin black). For these neurons, the distribution of directional signal indices (contra – ipsi/contra + ipsi; response epoch) was significantly shifted in the negative direction (Figure 5C; Wilcoxon; *p* < 0.01) and the number of neurons that fired more strongly for ipsilateral movement were in the majority on GO trials (Figure 5C; black bars; 0 vs. 21; chi-square = 20.8; *p* < 0.01). Significant differences between contralateral and ipsilateral GO trials appeared later in this population as compared to the one described previously, which appeared prior to execution of the movement (Figure 2A). Here, significant differences between these two trial types was not significant prior to port exit (100 ms epoch; *t*-test; *t*_(46)_ = 0.17; *p* = 0.86), but emerged in the 100 ms after (100 ms epoch; *t*-test; *t*_(46)_ = 2.46; *p* < 0.02). These results suggest that these neurons are more involved in inhibiting or allowing movement during its execution.

Surprisingly, the directional response was inverted during STOP trials and did not show the same flip-flop pattern of signaling one direction, and then the other, as described for neurons that fired more strongly under GO trials (Figures 2–3). Instead, there was a sharp phasic increase in activity during STOP trials in which the ultimate response was to be made in the contralateral direction (thick gray vs. thin gray). This difference was significantly different in the 100 ms after but not before (*t*-test; *t*_(46)_ = 0.04; *p* = 0.97) onset of the STOP cue (*t*-test; *t*_(46)_ = 3.62; *p* < 0.01). For STOP trials, the directional signal was significantly shifted in the positive direction (Figure 5D; Wilcoxon; *p* < 0.01) and roughly equal numbers of neurons fired more and less strongly for contralateral movement (Figure 5D; black bars; 9 vs. 11; chi-square = 0.18; *p* = 0.67).

**Figure 6:**
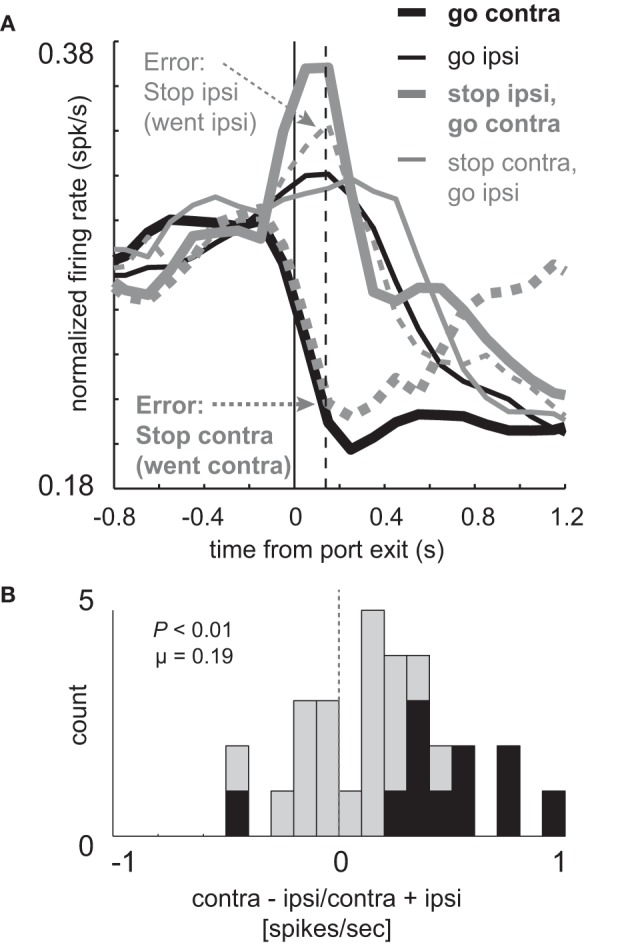
**Elevated firing under STOP trials is reduced during errors. (A)** Curves representing normalized population firing rate (*n* = 30) for neurons that showed elevated firing on GO compared to STOP trials for movements made in the contralateral direction. **(A,B)** All other conventions as in Figure 3. Vertical dashed line represents the time required to stop and redirect behavior (SCRT) as computed by taking the difference between correct STOP and GO trials during these sessions.

Like the previous population of neurons, activity of these neurons appears to be strongly related to the actual behavior that the rat performed, exhibiting directional signals during the response consistent with the movement that is being made. More specifically, these results suggest that these neurons might be involved in inhibiting ipsilateral movement during STOP trials and that perhaps, without adequate inhibition, rats were unable to stop the ongoing movement. To examine this possibility we plotted activity on error trials during sessions in which there was at least one STOP error in each direction. The results of this analysis are plotted in Figure 6.

On correct STOP-change trials, activity to the contralateral direction (Figure 6A; thick solid gray) rose and plateaued prior to the SCRT (Figure 6A; vertical dashed line; 140 ms), suggesting that activity was intimately related to cancelation of the ipsilateral response and that adequate firing is necessary to stop the unwanted response. If true, then this activity should be reduced or eliminated when rats were unable to stop the ongoing movement. Consistent with this hypothesis activity was reduced when the ipsilateral movement was not correctly inhibited (Figure 6A; thin dashed gray). In fact, activity was not statistically different from ipsilateral GO trials during the response epoch (Figure 6A; thin dashed gray vs. thin solid black; *t*-test; *t*_(45)_ = 5.04; *p* < 0.01). Further, the directional signal on unsuccessful STOP trials was inverted (Figure 6A; thick dashed gray vs. thin dashed gray) compared to GO trials (Figure 6A; black thick vs. thin) as illustrated by a significant positive shift in the directional signal index distribution (Figure 6B; Wilcoxon; *p* < 0.01) and the counts of neurons that fired significantly more strongly for contralateral movement were in the majority (1 vs. 10; chi-square = 7.2; *p* < 0.01).

Reduced activity on trials where the ipsilateral movement was not canceled suggests that these neurons play an inhibitory function. Further evidence for hypothesis comes from analysis of movement speed. If these neurons are involved in inhibiting unwanted movement in the ipsilateral direction then their activation should allow for faster contralateral responding. That is, stronger firing on STOP trials should reduce competition with neurons that signal contralateral movement.

**Figure 7:**
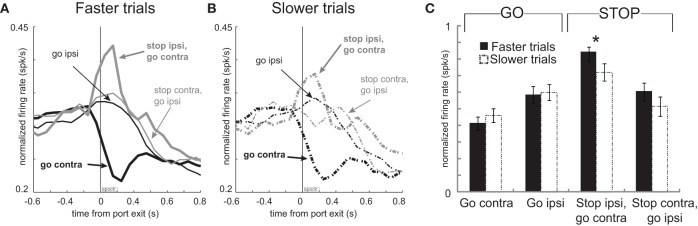
**Increased activity associated with faster movements on STOP trials. (A–C)** Curves representing normalized population firing rate during performance of each of the four conditions for neurons that showed elevated firing on GO compared to STOP trials for movements made in the contralateral direction. All conventions as in Figure 4.

To test this hypothesis, we again split trials in each session based on the speed of the response and replotted the activity for these neurons (Hanes and Schall, 1996). Faster and slower movement times for correct GO trials were 347 ms and 602 ms, respectively. For correct STOP trials, the average fast and slow movement times were 495 ms and 747 ms, respectively (*t*-test; *p*'s < 0.01). Consistent with our hypothesis, activity was significantly stronger immediately after port exit (200 ms) for STOP trials during which contralateral movement was significantly faster (Figures 7A and B thick gray solid vs. dashed; *t*-test; Figure 7C *t*_(92)_ = 2.4; *p* < 0.017). No other comparisons reached significance (Figure 7C; *t*-test; *p*'s > 0.05). Stronger firing when contralateral movements were faster combined with reduced firing on error trials during which rats incorrectly moved ipsilaterally (Figures 6A and B) suggests that phasic increases in firing of these neurons inhibit ipsilateral movement.

### Global stop signals in mDS

Thus far the effects that we have described in mDS are largely directional, with neurons firing more or less strongly for contralateral movement on unimpeded GO trials. The two populations that we have described appear to play a role in facilitating and inhibiting specific action in a directional manner. However, one can imagine that other neurons in the brain might be critical for stopping behavior in general, signaling for a “global” suppression of motor output, so that the correct response can more easily be generated. Both types of stop signals, global and action selective, are thought to exist in the brain (Greenhouse et al., 2012; Majid et al., 2012).

Here we ask if this global stop signal is present in mDS as defined by neurons that fire on STOP trials regardless of response direction. For this analysis we performed a 2-factor ANOVA with STOP and GO (trial-type), and contralateral and ipsilateral direction as factors. A global stop signal should show a main effect of trial-type with no interaction with direction.

**Figure 8:**
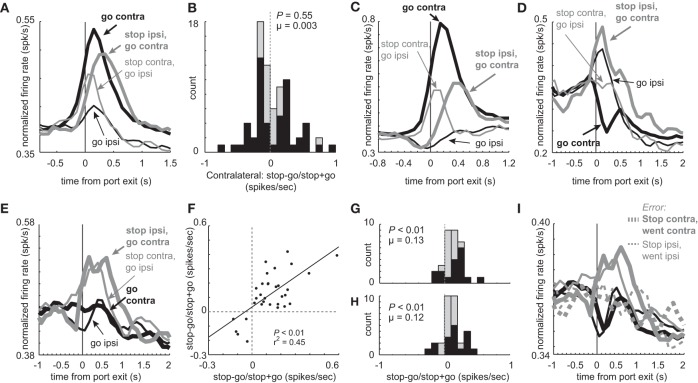
**Main and interaction effects in mDS. (A)** Average firing over time for the 76 neurons that show an interaction between direction (contra vs. ipsi) and trial-type (GO vs. STOP). **(B)** Distribution reflecting the difference between STOP and GO trials during contralateral movement during the response epoch for those neurons that showed a significant interaction between response direction and trial-type (GO vs. STOP). Black bars indicate neurons that fired more or less strongly on STOP vs. GO trials (*t*-test; *p* < 0.05). **(C)** Population of neurons that showed a significant interaction between direction and trial-type and showed significantly higher firing during GO trials in the contralateral direction (*t*-test; *p* < 0.05; i.e., black bars below zero in **A**). Thick and thin lines represent contralateral and ipsilateral direction, respectively. Black and gray represents GO and STOP trials, respectively. Activity is aligned to nose poke port exit. **(D)** Same as in **C** except for neurons that showed a significant interaction between direction and trial-type and showed significantly higher firing during STOP trials in the contralateral direction (*t*-test; *p* < 0.05; i.e., black bars above zero in **A**). **(E)** Curves represent normalized population firing rate for neurons that showed a main effect of trial-type with no interaction with direction in a 2 factor ANOVA (GO vs. STOP; contralateral vs. ipsilateral) during the response epoch. (**F–H**) Distributions of indices that compares activity on STOP vs. GO trials during the response epoch (stop – go/ stop + go) for contralateral and ipsilateral trials. Black bars represent within cell significant differences between STOP and GO (*t*-test; *p* > 0.05). **(F)** Correlation between values plotted in **G** and **H**. **(I)** Activity of neurons that showed a main effect of trial type with no interaction, taken from sessions where at least 1 STOP trial occurred in each direction. Black and gray represents GO and STOP trials, respectively. Solid and dashed lines represent correct and error trials, respectively.

Consistent with the notion that one of DS's key roles in behavior is to guide directionally specific motor output, many neurons showed a main effect of direction with no interaction of trial-type (*n* = 117; 27%). The majority of these neurons fired significantly more strongly for contralateral vs. ipsilateral GO movement (74 vs. 43; ANOVA; *p* < 0.05). In addition to these 117 directional neurons, other neurons (*n* = 76) showed a significant interaction between trial-type (STOP vs. GO) and direction (contra vs. ipsi) bringing the grand total of directionally tuned neurons during the response epoch to 144 (44%). The average population histogram for these ‘interaction’ neurons is illustrated in Figure 8A. Overall, activity was stronger for contralateral vs. ipsilateral movement, and was more pronounced for GO trials. At the single unit level, the number of neurons that fired more strongly for GO and STOP trials in the contralateral direction did not statistically differ. This is illustrated in the distribution in Figure 8B, which plots an index that measures higher (positive) or lower (negative) firing on STOP trials compared to GO trials in the contralateral direction (stop index = stop – go/stop + go; response epoch). The distribution appears to be bimodal and the counts of neurons showing stronger firing for GO (*n* = 26) and STOP (*n* = 25) trials were not significantly different (26 vs. 25; chi-square = 0.02; *p* = 0.90). The firing patterns of these neurons (Figures 8C and D), which are a subset of the neurons described in Figures 2A and 5A, are similar to what we have described in the previous sections.

Meanwhile, there were some neurons that showed characteristics consistent with a global stop signal. Of the 437 total neurons, 32 (7%) exhibited a main effect of type (STOP or GO) with no direction interaction. The average population histogram of all these neurons is plotted in Figure 8E. Overall, activity was significantly increased during STOP (gray) compared to GO trials (black) for both contralateral (thick) and ipsilateral (thin) movements. Of the 32 neurons, 27 fired significantly more strongly on STOP vs. GO trials whereas only 5 showed the opposite effect (chi-square = 15.0; *p* < 0.01). The stop – go/stop + go index was significantly shifted in the positive direction for both contralateral and ipsilateral movements (Figures 8G (contralateral) and H (ipsilateral); Wilcoxon; *p*'s < 0.01), and the number of neurons that fired significantly more strongly on STOP vs. GO trials was in the majority for both directions (Figures 8G and H; black bars; chi-square; *p*'s < 0.01). Finally, the two distributions were correlated (Figure 8F; *p* < 0.01; *r*^2^ = 0.45). These neurons are likely to be involved in globally stopping unwanted movement on STOP trials. Consistent with this hypothesis, activity was reduced on errant STOP trials (Figure 8I; dashed) and was not significantly different than activity observed on GO trials (response epoch; dashed gray vs. black; *t*-test; *t*_(59)_ = 0.98; *p* = 0.33).

### Classification of neurons based on waveform and spiking rates

Above we describe two main types of neurons based on their activity patterns. We found that some neurons fired more strongly for STOP over GO trials and others that fired more strongly for GO over STOP trials. Neurons in both groups appear to promote responding in the contralateral direction, one by modulating contralateral movement and the other by inhibiting ipsilateral movement. Although there is no perfect way to classify neurons based on waveform shape and firing characteristics, and attempts to do so often lead to debate and controversy, here we simply ask if neurons that exhibit these different activity patterns might show differential characteristics often used to define the two main types of striatal neurons: interneurons and medium spiny neurons (MSNs) (Taira and Georgopoulos, 1993; Kawaguchi et al., 1995; Mallet et al., 2005; Berke, 2008; Schmitzer-Torbert and Redish, 2008; Gage et al., 2010; Wiltschko et al., 2010; Vigneswaran et al., 2011). For this analysis we examined waveform duration, baseline firing and ISIs separately for cells exhibiting higher firing for GO trials and STOP trials. Waveforms were not inverted, baseline firing was taken during 1 s starting 2 s prior to trial initiation (nose poke) and the average ISI was taken during the entire recording session.

**Figure 9:**
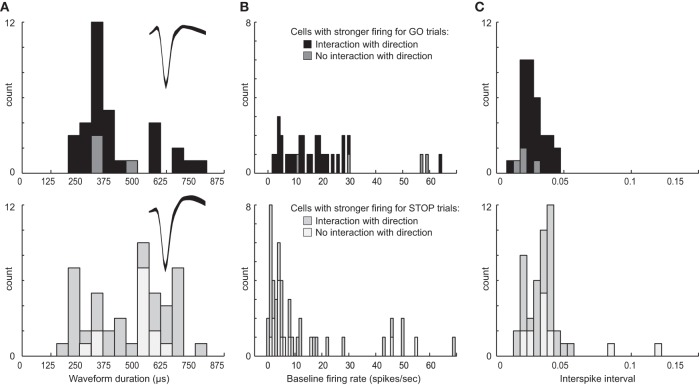
**Waveform and firing characteristics. (A)** Distribution of waveform durations as defined by the time between two maximum amplitudes for populations of neurons that showed stronger firing on contralateral GO trials (top) and STOP trials (bottom). Waveforms were not inverted. Insets plot the average waveform for these populations. **(B)** Distribution of average baseline firing rates taken during 1 s starting 2 s prior to trial initiation (nose poke). **(C)** Distribution of average interspike intervals taken during the entire recording session for each neuron. Lighter bars in each plot represent neurons that showed a main effect of trial-type with no interaction with response direction, firing more (top) or less (bottom) strongly for GO compared to STOP trials.

Interestingly, neurons that showed increased firing on GO trials tended to have higher baseline firing (Wilcoxon; *z* = 3.2; *p* < 0.01), shorter waveforms (Wilcoxon; *z* = 1.6; *p* = 0.11), and shorter ISIs (Wilcoxon; *z* = 3.1; *p* < 0.01) than did neurons that increased firing on contralateral STOP trials (Figure 9). Notably, this division was not entirely clear cut. That is, there was substantial overlap in all three measures. With that said, it appears from this analysis that at least a subset of neurons that exhibit these different activity patterns fall into different populations with the majority of neurons that fire significantly more strongly on contralateral GO trials showing characteristics more similar to interneurons and the majority of neurons that fired significantly more strongly on contralateral STOP trials showing characteristics more similar to MSNs. Firing of non-directional neurons that fired significantly differently for STOP vs. GO trials (i.e., global), also fell into these categories (Figure 9, lighter bars).

To further investigate the relationship between putative cell type and task-related firing patterns on GO and STOP trials, we went back to the original 437 neurons and categorized them based on baseline firing (>10 spikes/s), waveform duration (>500 μs) and ISI (>0.025), and asked how many neurons fired significantly more or less strongly on GO vs. STOP trials for movements made in the contralateral direction. Of the 437, 150 neurons showed characteristics common to be MSNs (Kim et al., 2009). Seventy neurons showed characteristics similar to interneurons. Of the 150 putative MSNs, 23(15%) fired significantly more strongly for STOP-change trials compared to GO trials correctly generated in the contralateral direction (*t*-test; *p* < 0.05). Only 3(2%) showed the opposite effect (chi-square = 15; *p* < 0.05). Of the 70 putative interneurons, the number of cells firing significantly more strongly for STOP or GO trials was not significantly different [8(11%) vs. 10 (14%); chi-square = 0.2; *p* = 0.6], however, the frequency of effects between populations was significantly different (23:3 vs. 8:10; chi-square = 7.9; *p* < 0.05). These results suggest that the majority of putative MSNs fired significantly more strongly under STOP trials compared to GO trials, whereas putative interneurons showed both activity patterns.

## Discussion

Few studies have examined neuronal activity in the context of response inhibition. Most of the work has been done in oculomotor countermanding tasks and/or have focused on frontal cortical regions (Hanes et al., 1998; Stuphorn et al., 2000; Ito et al., 2003; Stuphorn and Schall, 2006; Emeric et al., 2008). Here, we designed a novel task that allowed us to examine neural activity when rats had to inhibit a response that occurred on the large majority of trials and redirect behavior toward the opposite location. During performance of this task rats were less accurate and slower to respond on STOP trials. Slower movement speeds resulted from cancellation of an already initiated response (i.e., STOP cue was only signaled after response initiation).

Similar to classic STOP-signal tasks performed in rats and humans our rats had to inhibit an already initiated movement, however, unlike the majority of this work, our rats had to also redirect behavior in the opposite direction, more like so-called STOP-change tasks. During the design of this experiment, we suspected that rats might initiate corrective behaviors to help counteract incorrect movement and/or direct behavior toward reward, thus we decided to make redirection a requirement so that it could be better controlled when examining directionally tuned activity in mDS. Furthermore, since we required movements in both directions on all trial types, we could subtract latencies on STOP-change trials from latencies on GO trials to compute how much time the rat needed to stop and redirect behavior (SCRT), instead of estimating it with a stop-signal delay.

Pharmacological and lesion studies have implicated mDS in the control of behavior during performance of stop-signal tasks. Although rats with mDS lesions needed earlier warnings to be able to adequately inhibit movement as compared to controls, they were also slower on GO trials, suggesting that they not only had a deficit in response inhibition but also in general behavioral control (Eagle and Baunez, 2010). More recently, it has been suggested that dopamine in mDS may act to balance behavioral inhibition independent of behavioral activation. Manipulation of striatal D1 and D2 receptors, commonly associated with neurons that give rise to the direct and indirect pathways, influenced the imposition and speed of inhibition during stop-signal performance (Eagle et al., 2011). These results, combined with the electrophysiological results reported here, suggest than signaling of movement in mDS is complicated and that the ultimate output depends on the integration of several signals that promote or inhibit behavior as discussed below.

### Miscoding of direction and inhibition failure

In this study, we found a large number of mDS neurons that encoded response direction, increasing firing prior to the execution of the movement. Activity of these neurons related to movement speed in that higher activity was associated with faster movement times, suggesting that they facilitate behavior toward the contralateral direction. On STOP trials, during which rats were initially signaled to move contralaterally, activity quickly rose as during GO trials, then declined abruptly when the rat correctly refrained from making that movement. On error trials where the rat did not inhibit the contralateral response, activity continued to rise and was indistinguishable from correct GO trials. This suggests that when there was a miscoding of direction by these neurons, rats were unable to correctly inhibit responding in the contralateral direction.

On one hand, these neurons might be driving behavior through what has been described as the “go” or “direct” pathway in which activity from mDS directly modulates activity in globus pallidus internal segment (GPi) and substantia nigra pars reticulata (SNr), which are output structures in basal ganglia (Albin et al., 1989; Alexander and Crutcher, 1990; Mink and Thach, 1993; Maurice et al., 1999; Sato and Hikosaka, 2002; Kolomiets et al., 2003; Bromberg-Martin et al., 2010; Bryden et al., 2011; Maia and Frank, 2011). Recent work has shown that increases in firing of striatal interneurons as actions are being initiated, coincides with decreases in GP firing (Gage et al., 2010). Increased firing of mDS neurons would inhibit firing in these areas which would release downstream structures (e.g., superior colliculus) from inhibition to promote behavior (Felsen and Mainen, 2008).

On the other hand, these neurons might impact local circuits before influencing more motor-related downstream regions. Many of these neurons shared characteristics common to interneurons, having higher baseline firing, shorter waveforms and lower ISIs (Schmitzer-Torbert and Redish, 2008; Gage et al., 2010). Further, their activity patterns were similar to what has been described previously for interneurons in lateral parts of DS, firing more strongly for contralateral action at the time of the choice (Gage et al., 2010). Interneurons are thought to shape firing of MSNs in DS through feed-forward inhibition (Parthasarathy and Graybiel, 1997; Mallet et al., 2005; Gage et al., 2010). Thus, activity of these neurons might also shape behavior by impacting local circuits that then project downstream. Regardless of how these neurons ultimately impact behavior, their miscoding of direction was clearly related to failures in response inhibition.

### Inhibition of movement

Other neurons in mDS appeared to better serve an inhibitory function. The majority of these neurons increased firing on correctly performed STOP trials when the rat had to redirect to the contralateral direction. These neurons are most likely involved in inhibiting the ipsilateral response. Consistent with this notion, activity of these neurons was reduced when rats failed to inhibit the response, and higher firing was associated with faster contralateral responding on correct STOP trials. Interestingly, these same neurons decreased activity during contralateral GO trials, which is typically associated with the release of inhibition of downstream areas to allow for movement (Hikosaka et al., 2006; Bromberg-Martin et al., 2010). Thus, unlike the previous set of neurons, these neurons appear to inhibit ipsilateral movement and allow contralateral movement by inhibiting areas downstream.

Consistent with this hypothesis, these neurons shared firing and waveform characteristics that have been used to categorize neurons as MSNs. MSNs are thought to project out of the striatum to impact behavior via direct and indirect pathways through basal ganglia (Albin et al., 1989; Alexander and Crutcher, 1990; Mink and Thach, 1993; Maurice et al., 1999; Redgrave et al., 1999; Gurney et al., 2001; Sato and Hikosaka, 2002; Kolomiets et al., 2003; Hikosaka et al., 2006; Deniau et al., 2007; Bromberg-Martin et al., 2010; Bryden et al., 2011; Maia and Frank, 2011). Based on the relationship that these neurons have with movement speed and errant responses, we suspect that they must be part of the indirect pathway which projects to globus pallidus external (GPe) then to subthalamic nucleus (STN) before impacting SNr. Since GPe and STN are inhibitory and excitatory, respectively, excitation of mDS would increase activity in SNr, whereas inhibition would reduce it. Thus, increased activity in striatum would indirectly increase activity in SNr, which would subsequently inhibit downstream motor structures critical for controlling body movements in rats such as superior colliculus (Felsen and Mainen, 2008).

Interestingly, a smaller but significant number of neurons appeared to be related to inhibition of both contralateral and ipsilateral movement. These neurons might represent a global stop signal that inhibits movement in general, or at least, movements common to both directions. Inhibition during stop signal performance is thought to reflect both global inhibition and specific inhibition of undesirable movement (Greenhouse et al., 2012). Thus, this activity might be critical for inhibiting overall movement by shutting down all movement. Consistent with this hypothesis, depressed activity in these neurons was observed when response inhibition failed.

### Control of behavior

Patterns observed here, in mDS, resemble firing in primate oculomotor regions such as the frontal eye field (FEF) during performance of a countermanding task in which monkeys were signaled to make a saccade to the periphery by brief illumination of a visual stimulus (Hanes and Schall, 1996; Hanes et al., 1998; Schall et al., 2000; Stuphorn et al., 2000; Schall, 2001). During performance of this task, on 20% of trials, a stop signal (re-illumination of the fixation point) instructed the monkey to not make the instructed saccade and to remain fixating at a central location.

They found, as we have here, neurons related to generating and inhibiting behavior. Activity of many neurons was correlated with faster eye movements contralateral to the recording site. Other neurons fired more strongly on STOP trials when the monkey had to maintain fixation. From these studies it has been suggested that generation of movement results from the activity of motor-related neurons reaching some activation threshold at which point a movement is generated. The response that is made depends on what neurons cross threshold first. This process has been described as a race between two (or more) competing movement signals (Logan et al., 1984). In the oculomotor example, if the firing of neurons that generate eye movements crossed threshold before the competing signal to maintain fixation, then the eye movement was erroneously generated. Models such as the race model could explain the relationship between cells that promote contralateral movement and those that globally inhibit behavior in our task. That is, if movement cells reach threshold before the cells that shut it down, then the response would be erroneously generated.

However, such a model cannot explain the relationship between neurons that appear to selectively inhibit ipsilateral movement and those that promote behavior in the contralateral direction. Instead, their proposed functions would complement each other; with one driving behavior contralaterally while the other inhibiting ipsilateral movement. In fact, we suspect that at least some proportion of these two populations must be directly impacting each other. Most of the neurons that fired more strongly for contralateral GO trials showed firing and waveform characteristics more common to fast spiking interneurons (FSIs), whereas those neurons that fired more strongly for STOP trials, appeared to be putative MSNs. These two populations of neurons have opposite direction preferences and previous work has suggested that FSIs shape firing patterns of MSNs (Gage et al., 2010). This relationship is consistent with the observation that putative interneurons appear to encode direction prior to MSNs.

In conclusion, we show activity in mDS is related to both the promotion and inhibition of behavior. Reduced response inhibition signals and miscoding of directional information was correlated with poor performance. Against this backdrop we can better address what happens in several mental disorders where the ability to inhibit behavior is impaired. Deficits observed in certain disorders or after lesions might reflect abnormalities in one or both of these populations.

### Conflict of interest statement

The authors declare that the research was conducted in the absence of any commercial or financial relationships that could be construed as a potential conflict of interest.
